# Intra-mPFC injection of sodium butyrate promotes BDNF expression and ameliorates extinction recall impairment in an experimental paradigm of post-traumatic stress disorder

**DOI:** 10.22038/IJBMS.2022.65000.14312

**Published:** 2022-09

**Authors:** Ahmad Mohammadi-Farani, Sajad Fakhri, Cyrus Jalili, Zahra Samimi

**Affiliations:** 1 Medical Plants Research Center, Basic Health Sciences Institute, Shahrekord University of Medical Sciences, Shahrekord, Iran; 2 Department of Physiology and Pharmacology, School of medicine, Shahrekord University of Medical Sciences, Shahrekord, Iran; 3 Pharmaceutical Sciences Research Center, Health Institute, Kermanshah University of Medical Sciences, Kermanshah, Iran; 4 Medical Biology Research Center, Health Technology Institute, Kermanshah University of Medical Sciences, Kermanshah, Iran; 5 Department of Immunology, School of medicine, Kermanshah University of Medical Sciences, Kermanshah, Iran

**Keywords:** Brain-derived neurotrophic – factor, Extinction, Prefrontal cortex, Post-traumatic stress – disorder, Sodium butyrate

## Abstract

**Objective(s):**

Therapeutic strategies that facilitate extinction are promising in the treatment of post-traumatic stress disorder (PTSD). Brain-derived neurotrophic factor (BDNF) has a crucial role in neural plasticity, a process needed for the retention of fear extinction. In this study, we investigated the effects of local administration of a histone deacetylase (HDAC) inhibitor, sodium butyrate (NaBu), on BDNF transcription and behavioral markers of extinction in the single prolonged stress (SPS) model of PTSD.

**Materials and Methods:**

NaBu was infused into the infralimbic (IL) subregion of the medial prefrontal cortex (mPFC) of male rats. The freezing response was recorded as the criterion to assess fear strength on the day of extinction as well as 24 hr later in the retention test. Other behavioral tests were also measured to evaluate the anxiety level, locomotor activity, and working memory on the retention day. HDAC activity and BDNF mRNA expression were evaluated after the behavioral experiments.

**Results:**

NaBu facilitated the recall of fear extinction in SPS rats (*P*<0.0001). SPS rats had higher HDAC activity (*P*<0.0001) and lower BDNF expression (*P*<0.05) than non-SPS animals. Also, anxiety was higher in the SPS group (*P*<0.0001), but locomotor activity (*P*=0.61) and working memory (P=0.36) were not different between SPS and Non-SPS groups.

**Conclusion:**

Our findings provide evidence that the mechanism of action of NaBu in the improvement of extinction recall is mediated, in part, by enhancing histone acetylation and reviving BDNF expression in IL.

## Introduction

Post-traumatic stress disorder (PTSD) is a debilitating stress-related disorder that is characterized by an exaggerated fearful response to reminders of a traumatic event (1). Single prolonged stress (SPS) is a widely used rodent model of PTSD that recapitulates many of the neurobiological and behavioral alterations observed in PTSD patients (2). It is shown that SPS rats have deficits in retention of extinct fearful memories acquired in classical conditioning paradigms (3). Clinical studies have found the same maladaptation in humans (4-7). In conditioning paradigms, subjects learn, through repeated exposure, that a neutral or conditioned stimulus (CS) is followed by an aversive or unconditioned stimulus (US). Thus, they show a fearful response, like freezing, when CS is used alone. The fearful response will gradually fade if CS is repeatedly presented without US, a process which is called extinction (8). Extinction is a popular measure for investigation of the neurobiology of PTSD in preclinical studies (9). It is found that some PTSD symptoms occur when there are deficits in certain neural networks responsible for extinction impairments (10). Three cerebral areas, including the Medial Prefrontal Cortex (mPFC), amygdala, and hippocampus are key regulatory regions for conditioning and extinction in the brain (11). According to a well-studied model, some featured characteristics of PTSD, like exaggerated startle responses or increased freezing behavior are due to reduced amygdala inhibition by mPFC (12-16). Two sub-regions of mPFC are found to be involved in the control of fear behavior. The infralimbic (IL) division has a suppressive role in the expression of fear responses through inhibition of the central nucleus of the amygdala, whereas the prelimbic (PL) division seems to enhance the expression of fear memory, presumably, by activating neurons in the basal amygdala (17-19). At molecular levels, changes in behavioral parameters stem from the effects of stress on synaptic structure and function (20). Previous studies show that for the proper establishment of extinction memory, neural plasticity is a prerequisite in mPFC (9, 21) and basolateral amygdala (22). Synaptic plasticity is a process that changes the connective strength between two synapses (23). Neurotrophins are a family of proteins that, among other factors, play important roles in the plasticity of synaptic transmission. The most related neurotrophin studied so far, is the brain-derived neurotrophic factor (BDNF). The role of BDNF in the extinction of fear memories is reviewed elsewhere (24). Bredy *et al*. (2007) found that extinction is accompanied by increased expression of BDNF mRNA in the PFC (25). The role of BDNF in the prefrontal lobe is further confirmed by the results of a recent study that showed that infusion of proBDNF into the IL prior to extinction training enhances the learning of extinction in a rat model of conditioning (26).

Histone acetylation plays an important role in the control of BDNF gene transcription. Histones are a group of chromatin proteins that have five major families: H1/H5, H2A, H2B, H3, and H4. Alterations of the acetylated state in certain histones are crucial in controlling the transcription of some eukaryotic genes. Histone acetylation or deacetylation is controlled by histone acetyltransferases (HATs) and histone deacetylases (HDACs), respectively (27). Histone deacetylase inhibitors like trichostatin A, sodium butyrate (NaBu), and valproic acid (VPA) are widely used in conditioning and extinction experiments (25, 28-30). Systemic administration of valproic acid has induced BDNF mRNA levels within mPFC after an extinction procedure. This was associated with enhanced acetylation of histone 4 around the promotor 4 of the gene (25). Exposure of rats to the SPS protocol also modulates histone acetylation and changes BDNF transcription in the hippocampus of conditioned rats (31, 32). Based on the above, we hypothesized that transcriptional changes in the BDNF gene that are due to modification of histone acetylation in mPFC might be an underlying cause for extinction deficits in PTSD subjects. Therefore, in the current study, we examined the effects of bilateral infusion of NaBu into the IL of SPS rats, on extinction learning and extinction recall in a cued fear conditioning model. 

## Materials and Methods

***Animals***

All experiments were carried out using male Wistar rats (7–10 weeks old, 230–260 g body weight). Animals were housed at a controlled temperature (22 ± 2 °C) and on a 12/12-hr light/dark cycle. Behavioral tests were performed between 08:00 am and 1:00 pm. food and tap water were provided *ad libitum*. Animals were handled according to the National Institutes of Health Guide for the Care and Use of Laboratory Animals (8th edition. Washington DC, National Academies Press, US).

***Drugs***

Sodium butyrate was purchased from sigma- Aldrich (Sigma-Aldrich, Taufkirchen, Germany; 50, 250 mM). It was dissolved and diluted in 0.9% saline solution and injected in a volume of 0.5 µl per side with an infusion rate of 0.5 µl/min. Doses were selected based on previous research (33, 34) and a pilot study in our laboratory. 

***Experimental groups***

**Figure 1:**
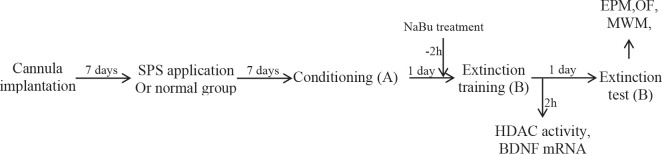
Timeline of the procedures. A; Context A, B; Context B, EPM; Elevated plus maze, NaBu; Sodium Butyrate, MWM; Morris water maze, OF; Open field, SPS; Single prolonged stress

Six groups of rats were used in our experiments. Three groups (n = 16) underwent the SPS procedure (SPS groups) and received different doses (0, 50, and 250 mM) of NaBu before the training session (Figure 1). After training, a subset of rats (n = 8), for each group, were sacrificed and used in the molecular experiments. The rest of the rats were tested through more behavioral procedures on the extinction day. Three groups of rats (n = 16) were non-SPS groups that were treated similarly to the SPS groups except that they were not exposed to the SPS procedure.

***Single prolonged stress procedure***

SPS was performed according to the procedure described above (35). It began with a 2-hr restraining period in which rats were immobilized for 2 hr in a clear polyethylene cone. Next, animals were immediately put into a pool of water (circular, 60 cm diameter, 22 °C) and forced to swim for 20 min. After a 15-min rest interval, rats were exposed to diethyl ether until they briefly lost their consciousness. Finally, they were returned to their cages and remained undisturbed for 7 days before more tests (Figure 1).

***Stereotaxic surgery***

**Figure 2:**
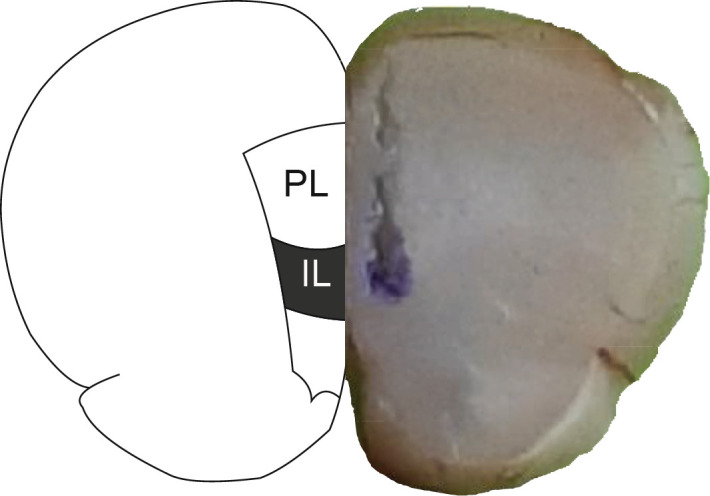
Cannula placement into the infralimbic division of mPFC. The infusion site of methylene blue is depicted in the right half of the representation

Surgery was performed as described elsewhere (36). Briefly, guide cannulas (stainless steel; 10 mm long, 22 gauges) were inserted bilaterally into the IL (AP: +3 mm, ML: ±0.8 mm, DV: -4 mm) (37) subregion of the brain while the animals were anesthetized with pentobarbital sodium solution (50 mg/kg, IP). The cannula was fixed to the skull, with dental cement, so that its tip was 1 mm above IL. After a 7–day period of recovery, the animals were used for further experiments (Figure 1). To confirm the precision of the position of the cannula, methylene blue (4%, 0.5 µl) was microinjected into the IL region at the end of the behavioral experiments. Brains were removed and kept in formalin (10%) for 3 days before they were sectioned and examined for cannula placement (Figure 2).

***Conditioning apparatus***

A fear conditioning apparatus, Ugo Basile (model 46002) controlled by AnyMaze software (Version 2.1; Ugo Basile), was used to study the cued fear in rats. It consisted of the main chamber, conditioning (context A), and a test (context B) box. Context A was a Plexiglas container (26×26×30 cm) that was settled on a stainless-steel electrified grid floor. The extinction training and extinction tests were carried out in a different box (context B) which had similar outside dimensions to context A but was changed internally by putting thin plastic inserts of different shapes and colors along its interior walls. Context B was further contrasted to the original training context (context A) by using a 0.5% peppermint solution for its aromatization. The main chamber had a white fluorescent lamp on top for the presentation of the conditioned stimulus (CS) and an LED lamp for providing a basal dim light.

***Conditioning and extinction protocols***

Conditioning took place in the context A of the apparatus. In the conditioning phase, animals received 7 pairings of the conditioned stimulus (CS), flashing house light (500 ms on/off, 8 lx, 30 sec), which was co-terminated by a foot-shock (0.7 mA, 2 s), as the unconditioned stimulus (US). The interval between each CS-US pairing was 2 min. One day later, NaBu was administered to the rats 2 hr before the extinction training. Extinction consisted of 8 cycles of CS (duration: 30 sec, interval: 90 sec) without presenting US in context B of the apparatus. More behavioral tests were performed after the extinction test was over. Twenty-four hours later, animals were tested for fear expression in context B while they were presented with a 2-min CS alone (Figure 1).

***Elevated plus maze test***

This is a plus-shaped maze made of two wooden open arms (60 × 12 ×1 cm) that are perpendicular to two closed arms (60 × 12 × 35cm). The apparatus was fixed 50 cm above the ground. The animals were put in the middle square of the crossing arms with their faces towards an open arm. Movements of the rats in the maze were recorded in a 10 min period by the EthoVision XT8 video tracking system (Noldus, Netherlands). The percentage of time spent in the open arms and the number of entries into the open arms were calculated by the software. These indices are decreased as the anxiety level increases in the rodents (38).

***Open field test***

The open field (OF) test is used to evaluate the locomotor activity and anxiety levels in the animals. The apparatus consists of a 75 cm × 75 cm × 40 cm enclosed square arena whose floor is divided into 25 equal squares (15 cm×15 cm). Animals are left separately in the field for a 5-min acclimatization period. After that, they were allowed for another 5 min free exploration during which the number of square crossings, as an index for locomotor activity, and the time spent in the center square, as an index for anxiety, were recorded (39). 

***Morris water maze test***

In this test, we used a black water tank (circular, diameter 120 cm, height 80 cm) as the water maze. The tank was filled with lukewarm tap water (23 ± 3 °C) to a height of 50 cm. There was a Plexiglas scape platform (circular, diameter 20 cm) in the center of an arbitrarily defined southwest quadrant of the pool that was submerged 2 cm below the surface of the water. Each rat underwent training and test sessions in the experiment. In the training session, the rat was released into the water in the opposite quadrant of the platform quadrant such that its head was towards the pool wall. During a 120 sec period, if rats could find the platform, they were allowed to stay for 10 sec and if they were not successful in locating it, they were gently guided to the platform and let stay on it for 10 sec. Next, the animals were towel-dried and kept in a warm cage until the test session. After 2 hr, the rats were placed in the same position of the pool and allowed to find the platform in 120 sec. The movement of the rats was recorded by a camera on top of the pool and the videos were analyzed by the EthoVision XT6 tracking system (Noldus Information Technology, Wageningen, Netherlands). Attenuation of the time needed to find the platform in the test session was considered an improvement in the working memory of the animals (40).

***Histone deacetylase activity measurement***

We evaluated the effects of NaBu treatment on HDAC activity 4 hr after drug treatment. Briefly, after decapitation, brains were removed, and the right and left IL were isolated on an ice-chilled petri dish. An ultrasonic homogenizer (Bandelin Electronic GmbH, Germany) was used to prepare homogenized tissues in 1 ml of 0.05 M phosphate buffer (pH=7). Samples were mixed to create 4 pools of 2 samples. A mixture was prepared by diluting the homogenates with an ice-cold lysis buffer (1:2 volume ratio, PH=7.4). The buffer contained: Tris–HCl (20 mM), EGTA (0.5 mM), sucrose (250 mM), KCl (10 mM), DTT (1 mM), EDTA (1 mM), okadaic acid (0.0001 mM), and PMSF (0.05 mM). After centrifugation of the mixture (20000 g for 5 min at 4 °C), the supernatant was removed and used for the HDAC test. A fluorometric HDAC Assay Kit (BioVision, USA) was used to determine the HDAC enzyme activity of the samples. First, assay buffer and fluorometric substrates were added to the wells in a 96-well plate and mixed. The plate was left in the incubator (37 °C) for a 30-min incubation period. Next, the developer solution was added to the mixture to stop the reaction. Thirty minutes later, a fluorescence plate reader (BioTek, USA) was used to read the samples in Ex/Em = 360/450 nm wavelength (Elsner *et al*. 2017).

***Real-time PCR***

Samples for the PCR were prepared 2 hr after the training test. Rats were decapitated and after removal of the brains, IL was isolated for PCR experiments. Four pools of 2 samples per pool were made from the homogenate samples described previously. Total RNA was extracted using the Tissue RNA Extraction Kit (Roche Applied Science, USA) as directed by the manufacturer’s instructions. We used a PrimeScript RT reagent Kit (Takara, Shiga, Japan) to make cDNAs from the RNA. The obtained cDNA was amplified using a StepOnePlus real-time PCR System (Applied Biosystems, USA). The internal standard was Glyceraldehyde-3-phosphate dehydrogenase (GAPDH). For BDNF target gene assessment, primer sequences were as follows: GAPDH fwd: 5´-tac-cag-ggc-tgc-ctt-ctc-ttg-3´, GAPDH rev: 5´-gga-tct-cgc-tcc-tgg-aag-atg-3´. BDNF fwd: 5´-tct-acg-aga-cca-agt-gta-atc-cc-3´, BDNF rev: 5´-tct-atc-ctt-atg-aac-cgc-cag-c-3´. Each reaction had a total volume of 25 µl that contained: primers; 1 µl/primer, Power SYBR Green PCR Master Mix 2X (applied biosystems, CA, USA); 12.5 µl, template; 1.5 µl and PCR grade water; 9 µl. Real-time PCR was performed with a holding stage (95⁰C for 10 min) that was followed by 40 cycles of denaturation 30 sec at 95 °C, annealing 30 sec at 56 °C, and extension 30 sec at 72 °C. 

***Statistical analysis***

For the extinction training tests, the two-way repeated measure (RM) ANOVA was used to evaluate the difference between the means among different groups. Data from the extinction test, EPM, OF and HDAC activity tests were compared by the two-way ANOVA with dose and stress as the main effects of comparison. A three-way analysis of variance was used to compare the working memory among different groups with treatment, stress, and training states as the main factors of comparison. We used GraphPad Prism 8.0.2 (GraphPad Software, Inc., CA, USA) to analyze data from the above experiments. In real-time PCR, we investigated the expression levels of BDNF using REST 2008 (V 2.0.7) as our software tool. The relative quantification method (2^-ΔΔCT^) was used to calculate the fold change expression ratio of BDNF transcription (41).

## Results

***Effect of SPS and NaBu on extinction learning in SPS-rats***

**Figure 3:**
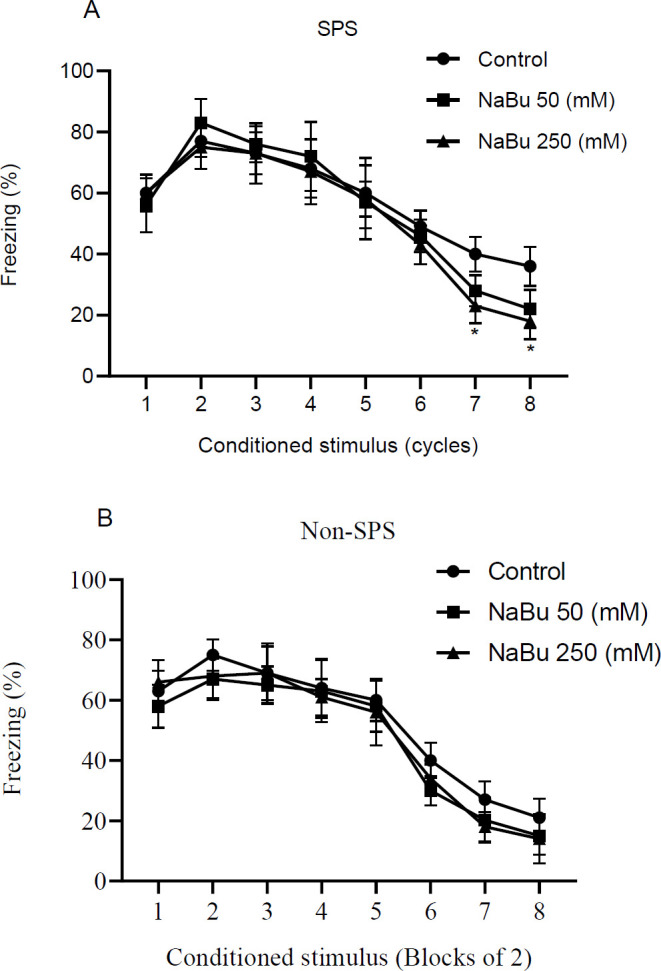
NaBu induced a significant enhancement of extinction on the training day in the SPS (A) but not the non-SPS (B) group. Percent freezing is depicted along cycles (8 CS-alone trials of 30 sec duration) of extinction training 1 day after the conditioning. The drug was injected 2 hr before the first cycle. Results are shown as mean ± SEM, n = 8. * *P*<0.05 relative to control in the same block

NaBu: Sodium butyrate; CS: Conditioned stimulus

One day after conditioning, extinction training was carried out in a different environment (context B) from the conditioning stage. The training was carried out in 8 cycles of US free trials. Results of a two-way repeated measure ANOVA showed that for both the SPS and Non-SPS animals, there was a significant difference among different cycles of training [SPS, F (7, 147) = 157.8, *P*<0.0001, n = 8; non-SPS, F (7, 147) = 124.6, *P*<0.0001, n = 8] (Figure 3). Different doses of the drug had also significant effects on the freezing behavior for SPS and non-SPS groups [SPS, F (2, 21) = 17.4, *P*<0.0001, n = 8; non-SPS, F (2, 21) = 14.3, *P*<0.0001, n = 8]. For the SPS group, there was significant interaction between cycle and stress [F (14, 147) = 2.3, *P*<0.05] while, the interaction was insignificant for the non-SPS group [F (14, 147) = 1.53, *P*=0.1]. *Post hoc* analysis revealed that in the SPS group, NaBu (at 250 mM) could significantly decrease freezing in the last two cycles of training compared with the control group on the same day (*P*<0.05 for both cycles). 

***Effect of SPS and NaBu on extinction recall in SPS-rats***

**Figure 4:**
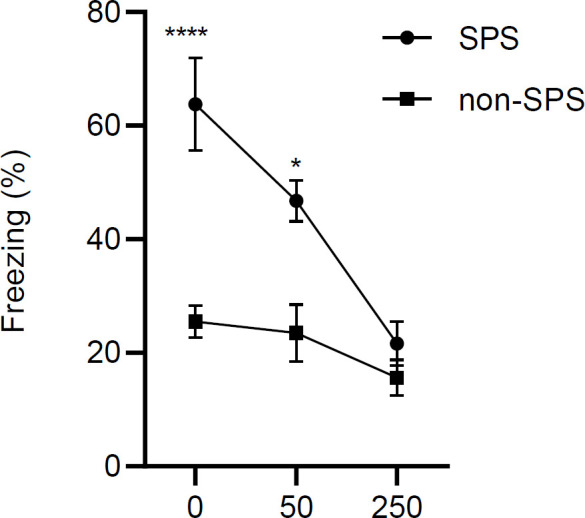
NaBu induced a significant enhancement of extinction on the retention test in the SPS group. Retention test was carried out 24 hr after the training procedure. It consisted of 1 trial of 120 sec in which animals received the CS anole. Results are shown as mean ± SEM, n = 8. * P<0.05 and **** *P*<0.0001 relative to SPS-control

NaBu: Sodium butyrate; CS: Conditioned stimulus

Two days after fear conditioning, rats were examined for freezing behavior in context B of the apparatus. The results showed that freezing was significantly higher in the SPS compared with non-SPS groups [F (1, 42) = 33.1, *P*<0.0001, n = 8] (Figure 4), and higher doses of the drug caused less freezing in the animals [F (2, 42) = 15.1, *P*<0.0001, n = 8]. There is also an interaction between dose and treatment in the extinction recall test [F (2, 42) = 5.6, *P*=0.006]. According to *post hoc* tests, in the SPS groups doses of 0 and 50 mg of NaBu had enhanced freezing compared with the non-SPS control group [ *P*<0.0001, *P*=0.02 respectively]. 

***Effect of SPS and NaBu on the elevated plus maze test in SPS rats***

**Figure 5:**
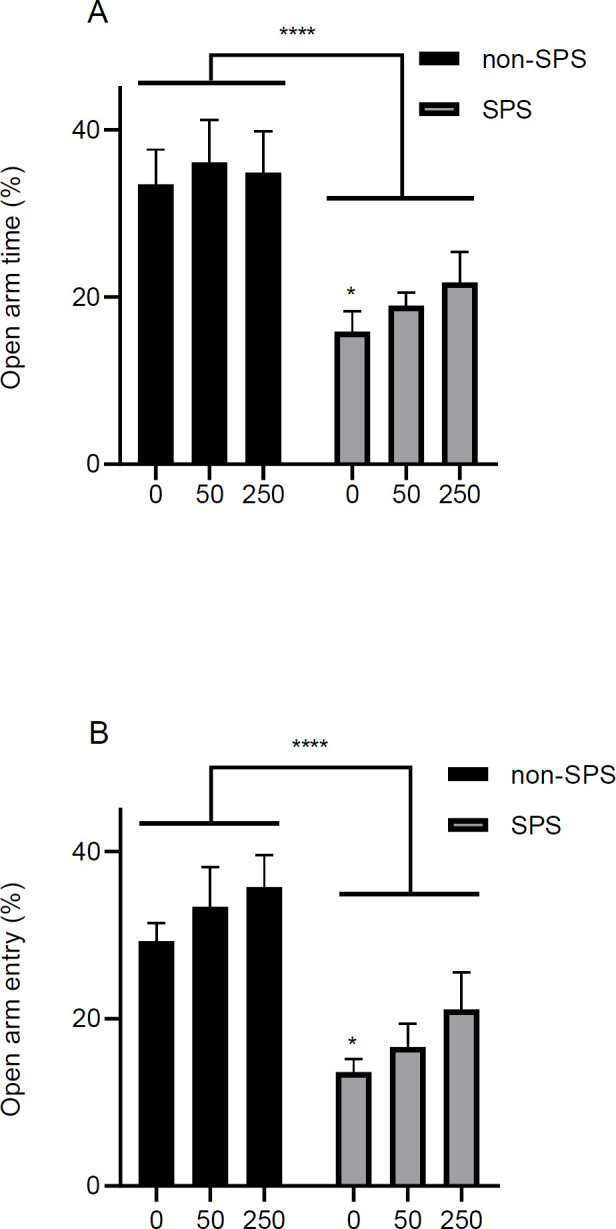
The amount of time spent in the open arms (A) and the number of entries into the open arms (B) of the EPM are represented in the graphs. The duration of the test was 10 min. Results are shown as mean ± SEM, n = 8. * *P*<0.05 compared with the non-SPS control, *** *P*<0.0001 for the main effect of stress

Levels of anxiety were measured by the EPM and OF tests. In the EPM test the amount of time that animals explored the open arms, in relation to the time spent on both arms, and the number of entries to the open arms, in relation to the total number of entries to the arms, were considered as the most relevant indices of anxiety. Analysis revealed that in terms of the time spent in the open arms and the number of entries into the open arms there was a significant main effect of stress [Time, F (1, 42) = 25.8, *P*<0.0001, n = 8; Entries, F (1, 42) = 30.8, *P*<0.0001, n = 8], but not a significant difference for the main effect of dose [Time, F (2, 42) = 0.49, *P*=0.61, n = 8; Entries, F (2, 42) = 2.0, *P*=0.14, n = 8], and no interaction between dose and stress [Time, F (2, 42) = 0.20, *P*=0.81, n = 8; Entries, F (2, 42) = 0.04, *P*=0.95, n = 8] (Figure 5). *Post hoc* analysis showed that there was a significant difference between the control groups in the stressed vs non-stressed rats for the exploration time [*P*=0.02] and the number of entries into the open arms [*P*=0.02]. 

***Effect of SPS and NaBu on the open field test in SPS rats***

**Figure 6.**
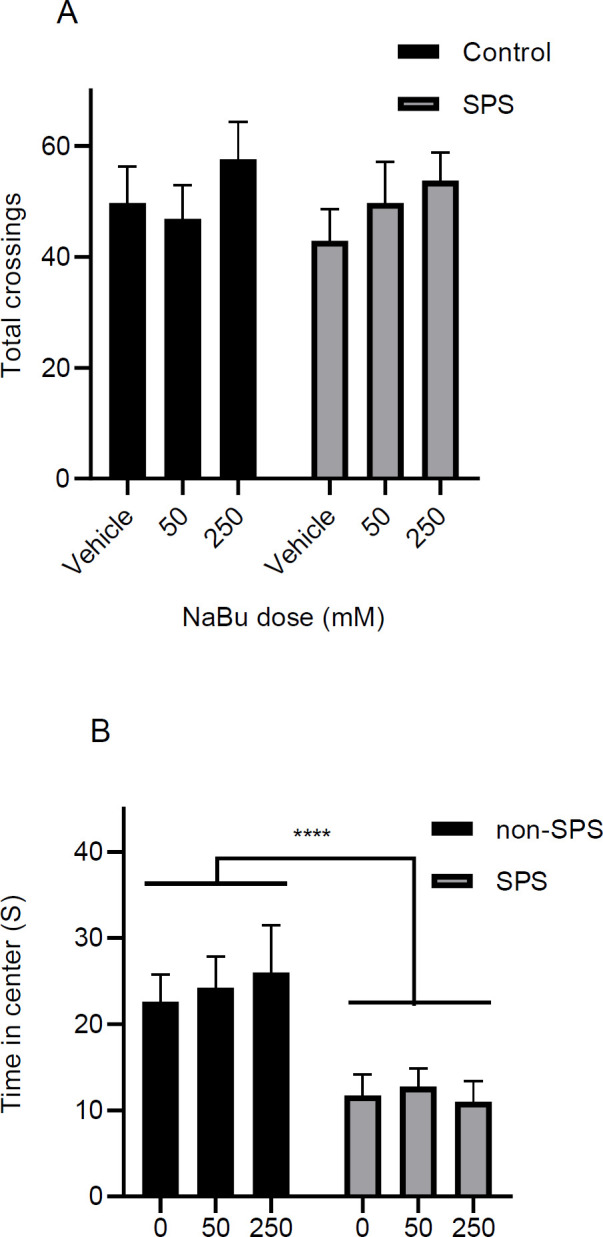
Effect of stress and treatment on the time spent in the center of the field (A) and total number of crossings (B) in the open field test are shown in the graph. Animals were free to explore the open field for 5 min. Results are shown as mean ± SEM, n = 8. *** *P*<0.0001 for the main effect of stress

As described before the test was performed to measure the locomotor activity and anxiety level in the animals (Figure 6). There was a significant difference for the main effect of stress in the amount of time spent in the center of the platform [F (1, 42) = 20.2, *P*<0.0001], but no significant effect for the main effect of dose [F (2, 42) = 0.09, *P*=0.90] or interaction [F (2, 42) = 0.21, *P*=0.80] was observed. Also, there was not a significant difference in the total number of crossings for the main effects of stress [F (1, 42) = 0.26, *P*=0.61], dose [F (2, 42) = 1.23, *P*=0.30] and the interaction between dose and stress [F (2, 42) = 0.31, *P*=0.73].

***Effect of SPS and NaBu on working memory in SPS rats***

**Figure 7:**
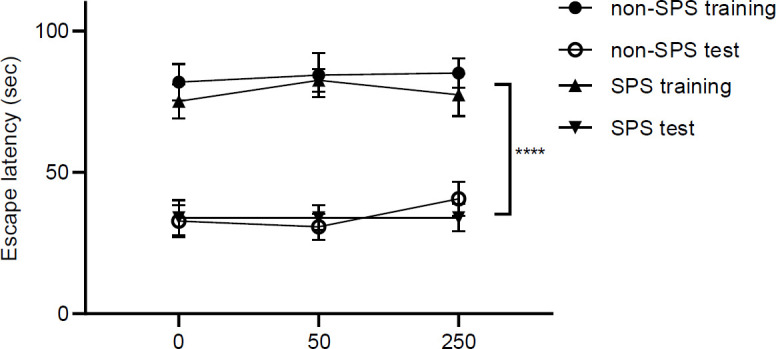
Latency to reach the platform in an MWM test is graphed for two groups (non-SPS and SPS) of rats receiving different doses of NaBu in the training and test sessions. Each session was 120 sec and the sessions were 2 hr apart. Results are shown as mean ± SEM, n = 8. **** *P*<0.0001 for the main effect of stress

MWM: Morris water maze; NaBu: Sodium butyrate;

In the MWM test, a three-way ANOVA test was used to evaluate the effects of stress, dose, and initial training on the latency to reach the platform. The results indicated that initial training of animals in the maze is the underlying cause for diminished escape latency of the animals [F (1, 84) = 193.4, *P*<0.0001, n = 8], but stress [F (1, 84) = 0.84, *P*=0.36, n = 8] or doses of NaBu [F (2, 84) = 0.33, *P*=0.71, n = 8] were not the determining main factors (Figure 7). The interaction among the three factors of stress, dose, and initial training was not significant [F (2, 84) = 0.08, *P*=0.91, n = 8].

***Effects of SPS and NaBu on HDAC activity***

**Figure 8:**
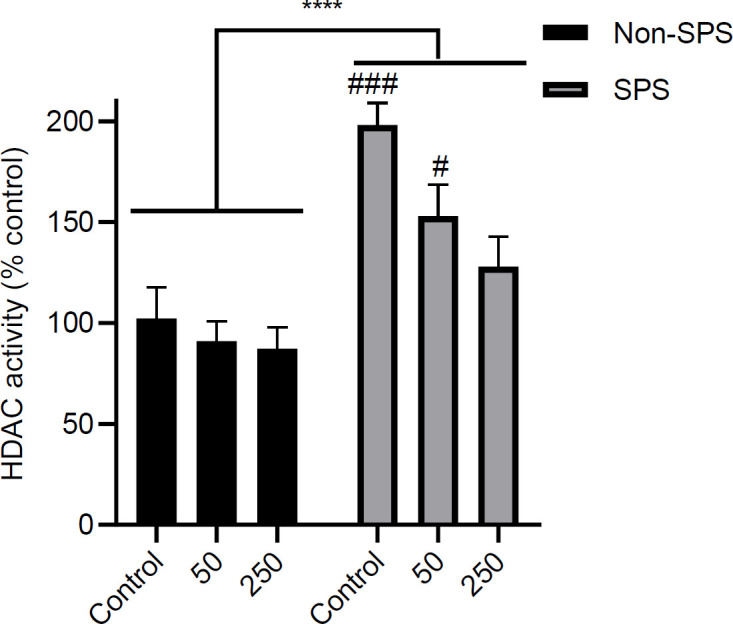
Effect of NaBu on HDAC activity in SPS and non-SPS groups. HDAC activity was measured in a separate cohort of animals immediately after the behavioral tests. Results are shown as mean ± SEM, n = 4 samples pooled from 2 rats/sample. **** *P*<0.0001 for the main effect of stress. ### *P*<0.001 and # *P*<0.05 compared with the non-SPS control

NaBu: Sodium butyrate; HDAC: Histone deacetylase; SPS: Single prolonged stress

The effects of NaBu treatment on HDAC activity in mPFC are depicted in Figure 8. Both stress [F (1, 18) = 38.43, *P*<0.0001, n = 4], and dose [F (2, 18) = 5.49, *P*=0.01, n = 4] were determining parameters for the amount of HDAC activity in IL. There was no interaction between the two factors [F (2, 18) = 2.26, *P*=0.13]. *Post hoc* analysis showed that NaBu could decrease HDAC activity in the SPS group in a dose-dependent manner compared with the non-SPS control group [non-SPS-control vs SPS-control, *P*<0.001; non-SPS-control vs SPS-50, *P*<0.05; non-SPS-control vs SPS-250, *P*=0.95). 

***Effect of SPS and NaBu on BDNF gene expression in SPS rats***

**Figure 9:**
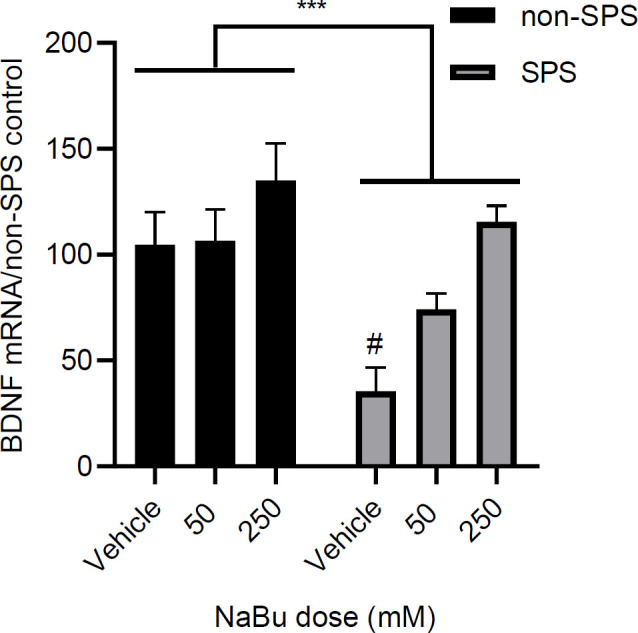
Relative quantitative plot for the effect of SPS exposure and NaBu treatment on BDNF transcription in mPFC. Results are shown as mean ± SEM, n = 4 samples pooled from 2 rats/sample. *** *P*<0.001 for the main effect of stress. # *P*<0.05 compared with the vehicle-treated non-SPS

SPS: Single prolonged stress; BDNF: Brain-derived neurotrophic factor; NaBu: Sodium butyrate; mPFC: Medial prefrontal cortex

Exposure to stress resulted in a significant reduction of BDNF transcription is SPS rats [F (1, 18) = 14.62, *P*=0.001, n = 4]. Treatment with NaBu enhanced BDNF expression [F (2, 18) = 9.31, *P*=0.001, n = 4], but there was no interaction between stress and treatment [F (2, 18) = 2, *P*=0.16, n = 4] (Figure 9). Data obtained from these studies will help us better interpret the results of the behavioral experiments and provide evidence for the probable mechanisms of stress in the neurobiology of PTSD. 

## Discussion

The purpose of this study was to evaluate the effect of NaBu on HDAC activity and BDNF expression in mPFC and to find its possible effects on the extinction process in rats. Our main findings are (1) Local pre-extinction treatment with NaBu facilitates extinction learning in SPS-rats as opposed to the non-SPS group in a cued conditioning model, (2) NaBu reverses the impairment of extinction recall in SPS-exposed animals in a dose-dependent way, (3) the expression of BDNF mRNA is increased in SPS rats in response to NaBu administration.

Impairment of inhibitory learning is a central dysfunctional element in the neurobiology of PTSD. It means that PTSD patients have difficulty learning to dissociate certain reminding stimuli with a past traumatizing event (42). This deficiency is mostly investigated in extinction-related studies in preclinical settings (7). Single prolonged stress (SPS) is a widely used animal model to study PTSD. In this study, we have investigated the effects of pre-extinction IL microinjection of a histone deacetylase inhibitor (NaBu) on behavioral responses and molecular modifications in the region. Previous studies show that enhancement of histone acetylation in the PFC and amygdala are essential epigenetic regulators for a proper extinction process (43, 44). Further immunohistochemical research suggested that extinction learning activates neurons and increases acetyl H3/H4 expression in the IL region of mPFC (45). In accordance with these studies, our experiments show that microinjection of sodium butyrate into the IL has two main extinction-related behavioral consequences: (1) it enhances (at 250 mM) the extinction learning process in the SPS group, as evident from reduced freezing in the last two cycles of extinction training (Figure 3), and (2) it reverses the impairment of extinction recall one day after extinction training in a dose-dependent manner (Figure 4). At molecular levels, a dose-dependent pattern is seen in the inhibitory action of NaBu on HDAC activity (Figure 8) in the SPS group, which is supportive of an HDAC-dependent action of the drug. Interestingly, NaBu has no effect on extinction and does not change HDAC activity in the non-SPS (normal) group. Therefore, it seems that exposure to single prolonged stress is the underlying cause for these molecular and behavioral changes. It is widely shown that systemic administration of some HDAC inhibitors like sodium butyrate (30), valproic acid (25, 28, 46), vorinostat (47), RGFP963 (48), and MS-275 (46) have enhancing effects on the learning or retention of extinction memory in normal rodents. The inconsistency between our results and these observations may be due to different routes of drug administration. In systemic treatments, multiple brain areas are exposed and regarding the complex nature of extinction memory, the interplay of multiple cerebral nuclei ultimately determines the outcome. However, we observed a declining trend in the freezing time in the recall test of the non-SPS group (Figure 4) which was associated with a similar trend in HDAC activity (Figure 8). Therefore, it is also probable that we could see significant effects if higher doses of NaBu were used in the non-SPS rats.

The determinant role of BDNF in the extinction of fearful memories is shown in several studies. These studies have particularly focused on the BDNF effect in the hippocampus (49, 50), amygdala (51-53), and prefrontal cortex (54-56). A recent study shows that SPS rats have lower BDNF protein levels in their IL. This study also finds that infusion of BDNF into IL, 1 hr prior to extinction training, ameliorates the impaired extinction in the retention test (54). In concert with this study, our results indicate that BDNF gene transcripts are less expressed in the IL of the SPS group (Figure 9), and impairment of extinction is recovered at a dose that brings the transcript levels back to normal (Figures 3, 4). Regarding the influence of stress on BDNF gene transcription, Takei *et al.* (2001) have shown that exon-specific m-RNA levels of BDNF are increased after contextual fear conditioning in the hippocampus of SPS rats (32). These findings suggest that stress-induced transcriptional changes in the BDNF gene might be an underlying factor, and hence a good treatment target in patients with PTSD.

BDNF transcription is regulated by transcriptional factors and epigenetic mechanisms (57). As an epigenetic regulatory mechanism, acetylation of H4 in chromatins at BDNF promoter IV and its mRNA transcripts were increased in the prefrontal cortex when rats underwent the extinction protocol (25). We have found that contrary to the non-SPS group, BDNF transcription is significantly increased in the SPS group after NaBu treatment (Figure 9). In fact, it seems that treating animals with SPS has induced a state in which they have become more sensitive to the effects of the drug. These results collective with the results obtained from the enzymatic assessment (i.e., enhanced reduction of HDAC activity in the SPS group) imply that reduced BDNF transcription in the SPS group is secondary to enhanced deacetylation of BDNF-regulating histone sites. It appears that NaBu enhancement of extinction recall involves a reverse process in the IL.

Additional behavioral tests were performed to find if SPS and NaBu would affect other confounding animal behaviors. The results of the EPM in conjunction with the OF test disclose that there is a significant difference in the main effect of SPS-state in that the SPS group has higher anxiety scores in both tests (Figures 5, 6B). Other investigators have also reported the association between anxiety and SPS (58-64). Increased anxiety is a common complication in many patients with PTSD (65). In preclinical settings, it induces a sustained defensive state that is manifested as anxiety-like behaviors in different models (66). The result of the OF test also shows that the overall activity of the rats in the SPS group is not different from the non-SPS one (Figure 6A), which means that the differences reported in the EPM or extinction tests are not confounded by the locomotor activity of the animals. 

Working memory is a cognitive process that is one of the main functions of mPFC (67). It is believed that working memory and extinction have shared neurocircuitry in the brain (68, 69) and most probably, mPFC-hippocampus connectivity is involved (70). Therefore, we assumed that the effect of SPS or drug on the working memory might be similar to what was seen in the extinction test. The MWM test reveals that SPS exposure and dose of NaBu do not change the working memory effect (Figure 7). It is concluded that SPS and drug treatment have exclusive effects on fear memory traces and have no effects on working memory circuits. 

## Conclusion

The results of the current investigation provide evidence that SPS-induced impaired extinction retention is accompanied by higher HDAC activity and reduced BDNF transcription in IL. This impaired retention is rescued by local pre-extinction administration of NaBu that leads to the reversal of HDAC activity and BDNF transcription. It is postulated that increased acetylation of histones near BDNF gene sequences, and the resultant up-regulation of BDNF expression, are the basis of NaBu-induced enhancement of extinction retention in PTSD rats. 

## Authors’ Contributions

AMF and SF Designed the experiments; AMF and ZS Performed experiments and collected data; AMF and CJ Discussed the results and strategy; AMF Supervised, directed, and managed the study; AMF, SF, CJ, and ZS Approved the final version to be published.

## Conflicts of Interest

On behalf of all authors, the corresponding author states that there are no conflicts of interest.
